# Effect of the Technological Parameters of Milling on Residual Stress in the Surface Layer of Thin-Walled Plates

**DOI:** 10.3390/ma17051193

**Published:** 2024-03-04

**Authors:** Magdalena Zawada-Michałowska, Paweł Pieśko, Grażyna Mrówka-Nowotnik, Andrzej Nowotnik, Stanisław Legutko

**Affiliations:** 1 Faculty of Mechanical Engineering, Lublin University of Technology, ul. Nadbystrzycka 38D, 20-618 Lublin, Poland; p.piesko@pollub.pl; 2 Faculty of Mechanical Engineering and Aeronautics, Rzeszow University of Technology, al. Powstańców Warszawy 12, 35-959 Rzeszów, Poland; mrowka@prz.edu.pl (G.M.-N.); nowotnik@prz.edu.pl (A.N.); 3 Faculty of Mechanical Engineering, Poznan University of Technology, pl. Marii Skłodowskiej-Curie 5, 60-965 Poznań, Poland; stanislaw.legutko@put.poznan.pl

**Keywords:** residual stress, aluminum alloy, milling, strain, thin-walled element, aviation

## Abstract

The production of thin-walled elements, especially those with large overall dimensions, poses numerous technological and operational problems. One of these problems relates to the machining-induced strain of such elements resulting from residual stress generated during the machining process. This study investigates the effect of the technological parameters of milling on residual stress in the surface layer of thin-walled plates made of aluminum alloy EN AW-2024 T351 for aerospace applications. The results have shown that residual stress increases with the cutting speed only to a certain point, reaching the maximum value at *v_c_* = 750 m/min. At a cutting speed *v_c_* = 900 m/min, residual stress significantly decreases, which probably results from the fact that the milling process has entered the High-Speed Cutting range, and this inference agrees with the results obtained for the cutting force component. Residual stress increases with the feed per tooth, while the relationship between residual stress and milling width is the same as that established for residual stress and variable cutting speed. Positive tensile stress is obtained in every tested case of the milling process. The results have also shown that the induced residual stress affects the strain of machined thin-walled parts, as proved by the strain results obtained for milled thin walls.

## 1. Introduction

A rapid development of machining processes in terms of product dimensional and shape accuracy has led to the wide application of these processes in various industrial branches, such as automotive, aviation, military, and electronics [1,2,3,4]. When it comes to the manufacture of thin-walled parts, the greatest problem is posed by strain generation during machining operations, particularly milling. Elastic strain leads to the formation of shape defects and reduced surface quality, while plastic strain induces residual stress that is difficult to remove from the surface layer of the workpiece, causing its permanent distortion [5,6,7,8]. 

Milling-induced residual stress has been investigated for years, both theoretically and experimentally [9]. This results from the fact that residual stress generation remains a vital yet unresolved industrial problem. It should be highlighted that residual stress plays an important role in critical aircraft components, particularly thin-walled parts [10]. It is estimated that aircraft manufacturers incur economic losses amounting to hundreds of millions of dollars (or even more) due to strain in these thin-walled components. To ensure production optimization, cost reduction, and improved aircraft operation safety, research should be conducted on residual stress and its root causes, as well as solutions for reducing this stress [11,12,13]. 

Residual stress generated during machining primarily results from cutting force and temperature. Cutting force predominantly depends on the cross-section of the cut layer, which means that it depends on the feed and depth of cut, as well as on other technological parameters such as cutting width. On the other hand, temperature is inextricably linked to cutting speed and cooling conditions. It is worth emphasizing that compressive stress in the surface layer is induced by cutting force, while tensile stress is generated by temperature. They also depend on other factors, including structural modifications and the resulting changes in material volume [14,15,16]. Taking the above factors into consideration, once can distinguish two main models describing residual stress generation in the surface layer of a material during machining [17]:

- Mechanical model which is based on the assumption that the cutting process depends on cutting force and that compressive stress is induced in the surface layer, while tensile stress is induced in the core;
- Thermal model which focuses on only the cutting process, assuming that tensile stress is induced in the surface layer, while compressive stress is induced in the core.

In real process conditions, both models overlap, but their impact may vary depending on which of the two factors prevails. 

It must be highlighted that residual stress is one of the surface integrity features [18,19] and that it can have either a positive or negative effect on machine component operation. An equally important aspect is the distribution of residual stress and its magnitude, both having an impact on the strain, fatigue strength, and corrosion resistance of the workpiece. Compressive stress generally has a positive effect as it leads to increased strength and higher fatigue corrosion, as well as prevents microcrack formation. Tensile stress has the opposite effect because it reduces fatigue strength and may lead to intercrystalline corrosion [20,21,22,23].

The generation of residual stress on the workpiece surface during cutting is a very complex process due to strain inhomogeneity, a high temperature gradient, and phase transition. When yield stress is exceeded, the workpiece undergoes permanent deformation both on its surface and deep inside. The generation of residual stress during cutting processes depends on many different factors, such as technological parameters, cutting tool geometry and wear, as well as the properties of workpiece material [24,25,26,27]. 

One study [28] proposed a mathematical model for describing the relationship between curved surface residual stress and a new parameter called Undeformed Chip Volume, which depended on the depth of cut, tool radius, feed per tooth, and cutting width. Another study [29] showed that while the above approach assumed that the depth of cut, feed per tooth, and cutting width had the same impact on residual stress, this assumption did not, however, show agreement with most experimental results. Referring to the former of the above studies [28], it should be stressed that the results showed that residual stress in the milling of curved thin-walled parts was predominantly affected by the depth of cut and tool radius. It was concluded that the two factors had to be taken into consideration first for machining parameters optimization. 

Studies [30,31,32,33] demonstrated that the strain in the thin-walled part resulted not only from thermo-mechanical load impact and vibration but also from residual stress, which could be put under two groups. The first included machining-induced residual stress, while the other included residual stress induced by operations such as rolling or thermal treatment. The occurrence of residual stress is usually a complex phenomenon dependent on a number of thermal, mechanical, as well as structural factors. Residual stress is not only generated during the entire manufacturing cycle for a given part but also occurs during further operation of this part. As previously mentioned, prior to cutting, so-called initial residual stress is accumulated over the entire volume of the workpiece, this stress being the result of processes such as machining or thermal treatment. One study [23] showed that machining-induced residual stress was more important in terms of surface integrity because it was located in the surface layer. 

For the determination of the effects of selected factors on the nature and value of machining-induced residual stress for different materials, numerous analytical studies have been conducted that utilized various models and algorithms. Yi et al. [34] proposed a prediction model based on a genetic algorithm and BP neural network for the determination of milling-induced residual stress depending on milling parameters. It was found that milling-induced residual stress varied considerably with the penetration depth of the tool, occurring at 0.12 mm. Shan et al. [35] proposed a model for the prediction of machining-induced residual stress in orthogonal cutting, considering mechanical and thermal residual stress. The proposed model was based on the Johnson–Cook constitutive model, contact mechanics, and slip line theory. It was confirmed that machining-induced residual stress increased with cutting force and temperature. Cheng et al. [36] proposed a smart model for predicting residual stress that was based on Gaussian process regression and other machine learning algorithms. The model considered cutting parameters and different shapes of the specimen. The results demonstrated that the concave arc specimen was prone to compressive stress, while the convex arc specimen was prone to tensile stress, with residual stress being the largest for the latter specimen. It was also confirmed that the cutting speed, depth of cut, as well as the feed per tooth had a significant impact on residual stress. Zhang et al. [37] developed a 3D-coupled thermo-mechanical model for simulating model surface roughness and residual stress in the end milling of 5083 aluminum alloy. They used explicit dynamic analysis as an algorithm for the milling process, finding that the surface residual stress resulted from the compressed effect of the tool and that the tensile effect was a result of the thermal load, while the nature and value of residual stress varied depending on the employed technological parameters. The study also proposed dividing the machined surface into zones along the cutting depth direction, i.e., thermal–mechanical effect zone, mechanical effect zone, and no-effect zone. Chen et al. [38] proposed an algorithm combining grey relational analysis (GRA), back propagation neural network (BP), and nondominated sorting genetic algorithm-III (NSGA-III), which they used to investigate factors affecting machining-induced residual stress in the milling of Mg-Li alloy. The numerical model was validated in experiments, the results of which showed that surface tensile stress and subsurface compressive stress in Mg–Li alloy had a typical spoon-shaped distribution, proving the coupling interaction between the cutting force and temperature. Jiang et al. [39] proposed a model describing the relationship between cutting force, thermal load, and residual stress, concluding that the tool angle and feed per tooth had a significant impact on the cutting force, leading to residual stress generation. Xu et al. [40] investigated the effect of multi-pass cutting on residual stress. It was found that the stress which had accumulated inside the workpiece from previous passes had an impact on the cutting force, temperature, strain, and, consequently, on residual stress. It was concluded that unlike single-pass cutting, multi-pass cutting made it possible to reduce surface tensile stress and internal compressive stress. If the radial depth of the cut was higher than the depth of strain produced in the previous pass, the residual stress generated in the successive passes had similar values. This approach allowed for residual stress optimization by the multi-pass cutting strategy. Liu et al. [41] conducted a numerical and experimental investigation of tool geometry effect on residual stress in the orthogonal machining of Inconel 718. In the numerical analysis, they utilized the coupled Euler–Lagrange method to determine the effect of tool geometry on temperature, force, elastic strain, and residual stress. It was found that the tools with a negative rake angle and a sharp edge radius generated higher compressive stress on the workpiece surface than the tools with a positive rake angle and a higher edge radius. Meng et al. [42] conducted a numerical and experimental study of Ti6Al4V alloy, showing that residual stress could be controlled by employing appropriate values of prestress. In the study, the workpiece was stretched with prestress prior to milling. Zhou et al. [43] developed analytical models describing the generation of cutting force and temperature, which led to the creation of a residual stress generation model that was based on an elastic–plastic model and a relaxation procedure. The models were then validated in five-axis milling conducted with a torus-end mill. The developed mathematical models showed good agreement with the experimental results, demonstrating that residual stress increased with cutting speed. It was also found that residual stress depended on the edge angle and rake angle of the cutting tool. The values of these angles affected the cutting force and temperature, which resulted in changes in the values of residual stress. Jiang et al. [44] proposed a new empirical model for the superimposition of residual stress induced in milling, which considered the effects of the cutting force and temperature on residual stress separately. The numerical results demonstrated that compressive and tensile stress was induced by the cutting force, while tensile stress was predominantly generated by temperature. The study also focused on a mathematical model for the determination of residual stress depending on the cutting force-to-temperature ratio. It was found that the surface residual stress was induced when the force ratio was from 53% to 78% and when the force-to-temperature ratio ranged from 0.09 to 0.39. When the combination of cutting parameters had a greater impact on the cutting force than on the generated heat, the more favorable compressive stress was induced. 

It must be highlighted that some of these previous works were experimental studies. Ji et al. [45] studied the residual stress generated in the pocket milling of 2219 aluminum alloy. The results demonstrated that the depth of cut had the greatest impact on this stress. One study [17] investigated the effect of cutting speed on milling-induced residual stress for aluminum alloys 2024 and 7075. The milling process was conducted with variable cutting speeds reflecting conventional cutting and High-Speed Cutting (HSC). The results showed that surface tensile stress was higher in HSC than in conventional cutting. Weber et al. [24] found that an increased feed per tooth led to an increased penetration depth of residual stress and an increased depth of maximum compressive stress due to a higher load on the workpiece. As a result, there were more deformed areas, which led to deeper residual stress and the shift of the maximum stress moving deeper into the workpiece. In effect, the part milled with higher feed per tooth was deformed to a greater extent. An additional observation was that the repeatability of residual stress was higher in stable machining, whereas for unstable machining, it was much lower. Sivam at al. [46] studied residual stress in the dry milling of ZE41 magnesium alloy. The study investigated relationships between independent factors, such as feed per tooth, rotational speed, tool diameter and depth of cut, and dependent factors, including residual stress. The results of residual stress were, however, ambiguous, which was explained by the properties of the material under study. Nevertheless, the results confirmed that the cutting speed had a significant impact on the generated residual stress. Berry et al. [47] confirmed that an increased feed per tooth in combination with a reduced cutting speed resulted in higher compressive stress on the surface of the material. The fatigue test results unanimously showed that higher compressive stress led to improved fatigue life, yet this could induce fracture at the interface between the deformed and undeformed zones in the workpiece. Matuszak et al. [48] presented an analysis of the impact of High-Speed Machining of Ti-6Al-4V titanium alloy on the tool wear and surface layer properties, including residual stress and fatigue life. These researchers clearly stated that the cutting speed had a significant impact on generated residual stress.

As emphasized by Jiang et al. [9], the problem of cooling in milling processes is a very important aspect. Most previous studies showed that residual stress was lower when milling was conducted with cutting fluid than in dry milling. Nevertheless, it must be observed that differences occur resulting from the type of cooling applied. De Paula Oliveira et al. [49] investigated the effect of liquid nitrogen, minimum-quantity lubrication, and flood methods on residual stress in the cryogenic milling of Inconel 718. The results showed that the lowest residual stress was generated in milling by the liquid nitrogen method. Wika et al. [50] investigated supercritical carbon dioxide cooling with minimum-quantity lubrication (scCO_2_ + MQL) first and then with the use of flood coolant. The results showed no significant differences between residual stress induced in both milling processes. 

It should be stressed that the accuracy of the residual stress measurement depends on the repeatability of the results and the employed measuring method. One study [23] showed that apart from determining the effect of machining process parameters on residual stress, it was equally important to establish whether the obtained results were repeatable under the same machining conditions. Kuji et al. [13] reviewed methods for residual stress measurement by the 2D-XRD method and then compared these results with the results obtained by methods such as hole-drilling, slotting, sin2(ψ), and cos(α). The study was performed on specimens of AA7050-T7451 aluminum alloy, and the results showed agreement between the results obtained with the above methods. It should be noted that various methods of measuring residual stresses are used. Lostado Lorza et al. [51] used the strain gauge method to measure the residual stresses of welded joints.

The objective of this study is to investigate the effect of the technological parameters of milling on residual stress in the surface layer of milled thin-walled parts made of aluminum alloy EN AW-2024 T351. The approach adopted in this study results from the need for a detailed insight into both the milling process and the mechanisms of residual stress generation in the surface layer of machined materials as well as the residual stress-induced strain of thin-walled parts. Given that residual stress is more difficult to reduce in industry due to the overall dimensions of produced parts and high extra costs, it is therefore necessary to look for solutions that would help minimize strain induced during machining processes, as well as would make it possible to “control” the value and variation pattern of residual stress. 

## 2. Materials and Methods

**Figure 1:**
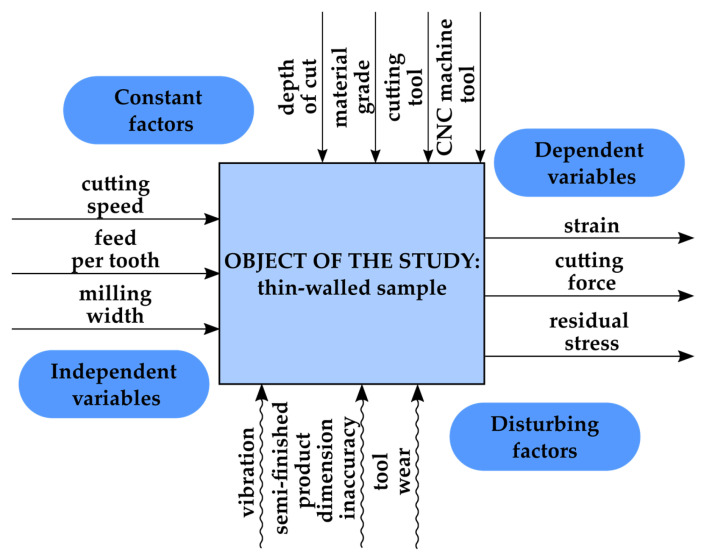
Research scheme.

Figure 1 shows the scheme of the research conducted on thin-walled samples. The independent variables included technological parameters such as cutting speed, feed per tooth, and milling width, while the dependent variables were residual stress, cutting force, and strain. The constant factors were the depth of cut, material grade (aluminum alloy EN AW-2024 T351), cutting tool, and CNC machine tool. The disturbing factors included vibration, semi-finished product dimensional inaccuracy, as well as tool wear.

**Figure 2:**
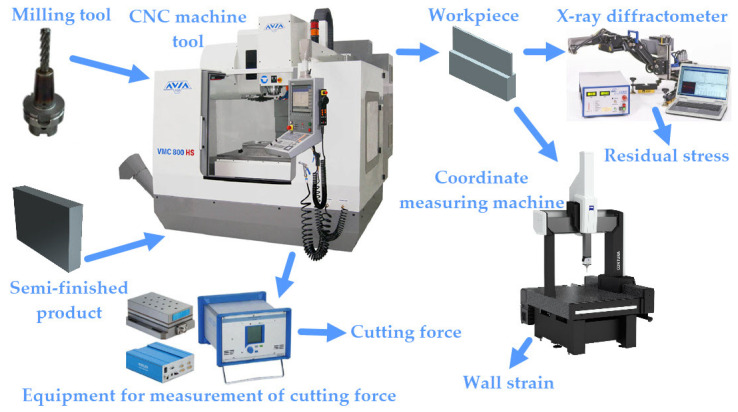
Experimental procedure.

The experimental procedure, test equipment, and measuring devices are shown in Figure 2.

The study was conducted on the aerospace aluminum alloy grade EN AW-2024 T351, which is widely used in the manufacture of thin-walled components, such as wing plating and aircraft fuselage. This material has good machinability (classified in Group II) and is characterized by high strength yet poor weldability, as well as reduced corrosion resistance. The chemical composition and selected properties of EN AW-2024 T351 are given in Table 1.

**Figure 3:**
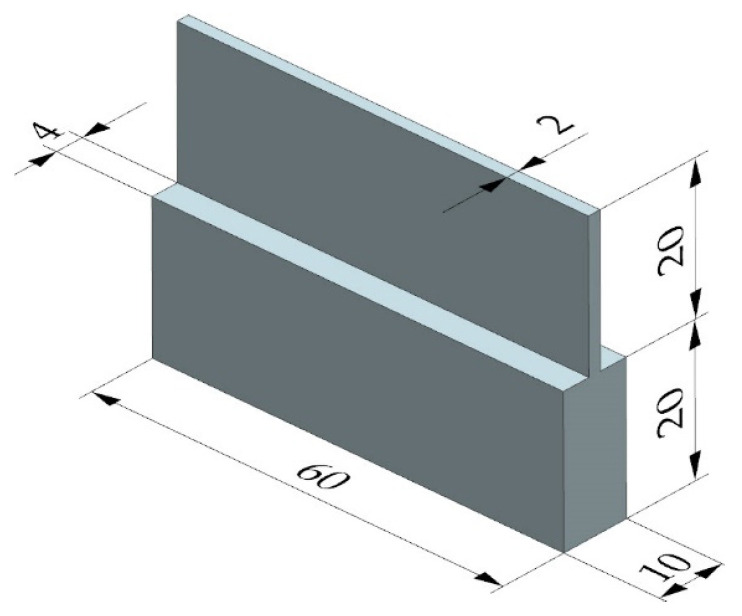
Geometrical dimensions of a thin-walled sample.

Thin-walled samples with overall dimensions of 60 × 10 × 40 mm were used in the experiments (Figure 3). The wall thickness after milling was 2 mm. Regarding the determination of the effect of the milling width *a_e_* on the generated residual stress, it must be noted that the wall thickness varied in the range of 1–6 mm, which corresponded to the milling width *a_e_* ranging from 4.5 to 2 mm. The semi-finished product was a rolled plate with a thickness of approximately 10 mm. 

The machining tests were conducted on the Avia VMC 800HS vertical machining center (Fabryka Obrabiarek Precyzyjnych AVIA S.A., Warsaw, Poland) provided with High-Speed Cutting and High-Performance Cutting functions. The tests were performed using a 12 mm diameter four-flute end mill from SGS (Wokingham, Berkshire, UK) with the symbol 44748. This tool is dedicated to full-depth machining of high and thin walls. Its basic geometric parameters are listed in Table 2. The end mill was mounted in an HSK63A shrink-fit toolholder (HAIMER GmbH, Igenhausen, Germany) first and then balanced in G2.5 class for a rotational speed of 25,000 rpm in compliance with [54], using the CIMAT CMT 15 V2N balancer (CIMAT Sp. z o.o., Bydgoszcz, Poland). During machining, the sample was clamped in a machine vice.

The tests were conducted for three cases of the milling process. In the first case, the cutting speed *v_c_* was variable, while the feed per tooth *f_z_* and the milling width *a_e_* were maintained constant. After that, the effect of the feed per tooth *f_z_* was verified, with the values of the cutting speed *v_c_* and milling width *a_e_* maintaining constant. For the last case, the cutting speed *v_c_* maintained constant along with the feed per tooth *f_z_*, while the milling width *a_e_* was variable. For each case, the depth of cut maintained constant at *a_p_* = 20 mm. The machining process was conducted in a single pass. It should be stressed that the feed direction of the cutting tool was perpendicular to the rolling direction of the semi-finished product. All milling parameters used in the experiments are listed in Table 3.

The adopted parameters and their ranges result from many years of experience of the authors, as well as the recommendations of the cutting tools’ manufacturers. The values of these parameters are commonly used in industrial practice for the machining of aluminum alloys. Smaller values of technological parameters correspond to conventional machining, and larger ones correspond to modern cutting techniques such as High-Speed Cutting.

**Figure 4:**
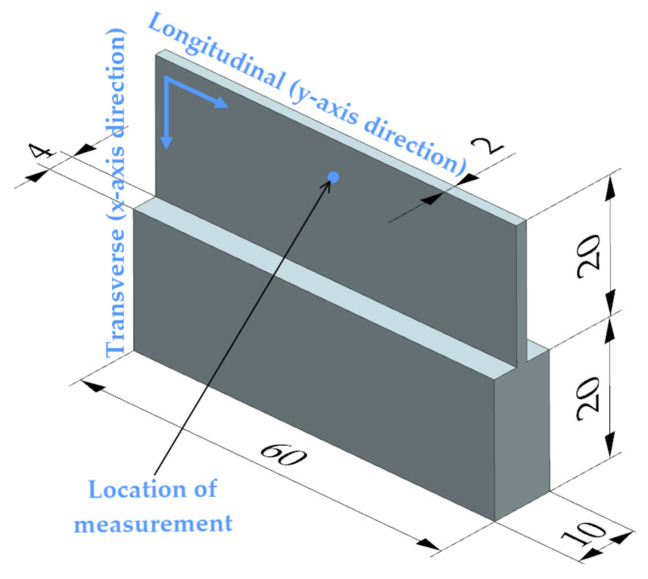
Model showing the location of residual stress measurement on a sample.

**Figure 5:**
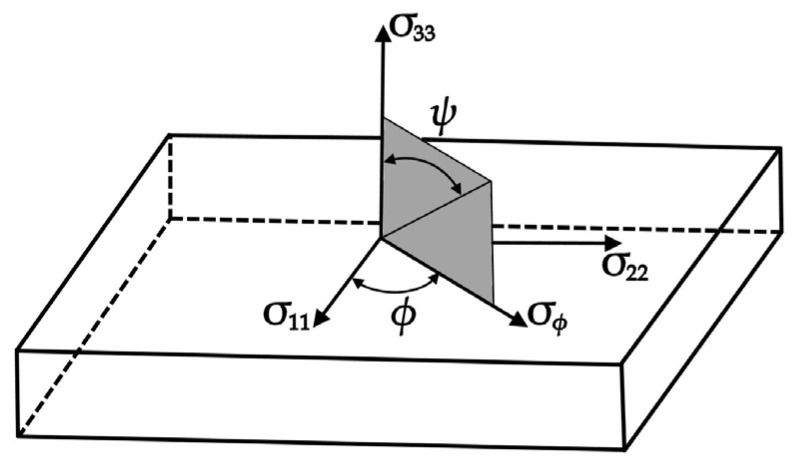
Main directions of the residual stress tensor *σ*, *φ*—angle between the main stress direction $\sigma_{11}\;$ and the selected measurement direction, *ψ*—angle between normal to the surface of the sample and normal to the family of planes {*hkl*}, $\sigma_{\phi }—$ component of the stress tensor in the direction of measurement.

XRD stress measurements were carried out on as-machined strips using the Proto-iXRD (Proto Manufacturing Ltd., LaSalle, ON, Canada) stress diffractometer by the sin2ψ method in compliance with the RUT’s good practice guide certified to [56]. A Cr K-α target tube was used with a wavelength of 2.1031 A°, an X-ray penetration depth of approximately 26 μm, and a round collimator of 2 mm in diameter. Measurements were made in the location marked in Figure 4 in order to determine stress magnitudes in both the longitudinal (y-axis) and transverse (x-axis) directions. Residual stress was calculated from the measured strains of (222) crystallographic planes at the 156.7° Bragg angle. Residual stress was measured according to [57]. For each point in both directions, measurements were made at eleven ψ off-set angles in the range of ±33°, where 10 acquisitions with a 3 s exposure time were made at each angle (Figure 5). The uncertainty of stress was calculated from the best fit to the sin^2^ plot. Residual stress was measured for each case of the milling process. In addition to that, residual stress was measured for the initial state, i.e., prior to machining (in the plots, it is marked as initial state). In this study, only residual stress was analyzed, excluding shear stress. 

The study also involved estimating the strain of the thin wall of the sample, which was quantified as a difference between the measured wall thickness *t_R_* and the nominal wall thickness value *t_N_* (1):

(1) $$∆t=t_{R}-t_{N}$$

The measurement was made using the Zeiss Contura 7/10/6 coordinate measuring machine (Zeiss, Oberkochen, Germany) equipped with an optical linear scanning head, LineScan 2–50.

To explain the mechanism of residual stress generation, the cutting force components (*Fx*, *Fy*, *Fz*) were measured with the Kistler 9257B piezoelectric actuator (Kistler, Winterthur, Germany) connected to a 5070A amplifier. The signal was then sent to a data acquisition module (DAQ 5697A) and processed using DynoWare v.2825A-01 software.

The microstructure of the alloy was examined in the cross-section of the sample transverse to the direction of deformation. The examination was performed using the Leica DMI-3000X light metallographic microscope (Danaher Corporation, WA, USA). The samples were cut using a precision cutting machine, Discotom–6 (Struers, Copenhagen, Denmark) and mounted in a bakelite (Struers, Copenhagen, Denmark). They were ground with SiC papers of 500, 800, 1000, and 1200 grit as well as polished using 3 and 1 µm diamond polycrystalline suspensions. Microstructural examination was performed on the samples that were polished and etched at room temperature using a modified Keller’s reagent: 2 cm^3^ HF + 3 cm^3^ HCl + 20 cm^3^ HNO_3_ + 175 cm^3^ H_2_O. Prior to the machining tests, the rolled EN AW-2024 T351 samples were also examined for their microstructure. The metallographic specimens were cut perpendicular to the direction of deformation. 

## 3. Results

**Figure 6:**
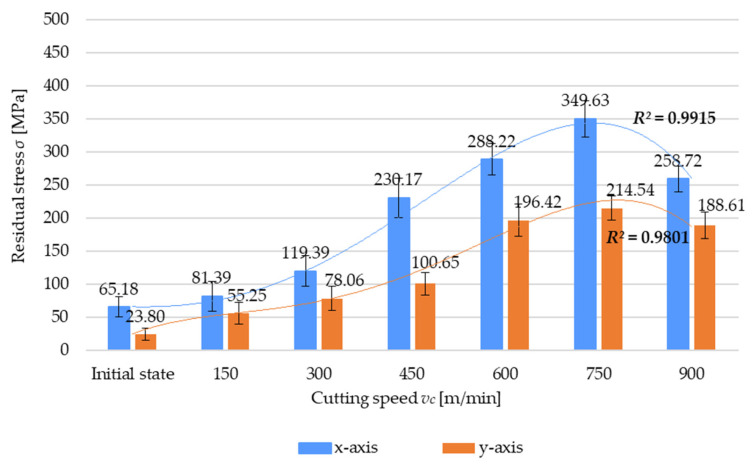
Residual stress *σ* versus cutting speed *v_c_*.

An analysis of the results presented in Figure 6 demonstrates that the residual stress *σ* increases with increasing the cutting speed *v_c_*. It reaches the maximum value at the cutting speed *v_c_* = 750 m/min and begins to decrease at *v_c_* = 900 m/min. This relationship can be observed for both the x- and y-directions of stress measurement. It should be noted that the difference between the maximum residual stress and minimum residual stress obtained from the initial test is well above 430% for the x-axis direction and 800% for the y-axis direction (relative to the initial state). It is also worth drawing attention to the sign of residual stress. For each tested case, residual stress has a positive sign, which indicates the presence of tensile stress and the dominance of the thermal mode of residual stress generation. The variation in residual stress as a function of the cutting speed for the x-axis (2) and the y-axis (3) direction was described by a fourth-degree polynomial:

*σ* = −0.9728(*v_c_*)^4^*k*_4_ + 8.7995(*v_c_*)^3^*k*_3_ − 9.1363(*v_c_*)^2^*k*_2_ − 6.98(*v_c_*)*k*_1_ + 73.863*k*_0_ (2)

*σ* = −1.4126(*v_c_*)^4^*k*_4_ + 19.467(*v_c_*)^3^*k*_3_ − 86.426(*v_c_*)^2^*k*_2_ + 173.83(*v_c_*)*k*_1_ − 80.987*k*_0_ (3)

where *k_i_*, representing the polynomial unit factors, corresponds to the unit ordinals:

$$k_{4}=1{\left[\frac{min}{m}\right]}^{4}\cdot MPa;\;k_{3}={1[\frac{min}{m}]}^{3}\cdot MPa;k_{2}=1\;{\left[\frac{min}{m}\right]}^{2}\cdot MPa;\;k_{1}=1\;\frac{min}{m}\cdot MPa;k_{0}=1\;MPa.$$

**Figure 7:**
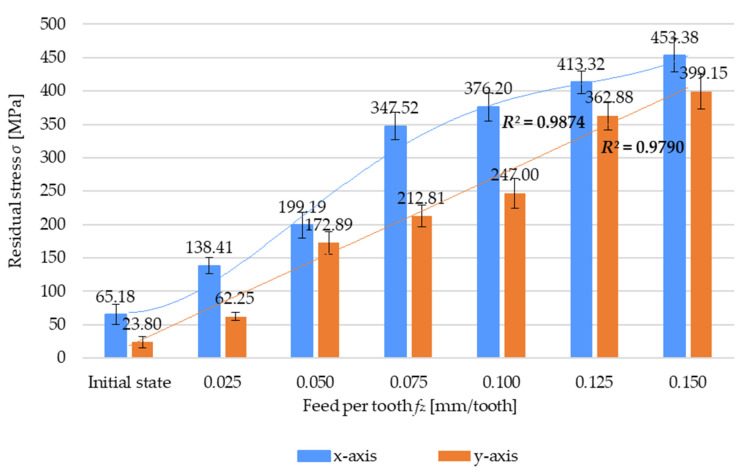
Residual stress *σ* versus feed per tooth *f_z_*.

Results of the residual stress *σ* as a function of the feed per tooth *f_z_* are presented in Figure 7. An analysis of the results demonstrates that residual stress increases with increasing the feed per tooth *f_z_* in both measurement directions. The residual stress for the highest tested feed per tooth value (*f_z_* = 0.15 mm/tooth) is almost 600% (in the x-axis direction) and 1600% (in the y-axis direction) higher than that obtained for the sample before machining. The same relationship can be observed regarding the residual stress sign because this case also shows the presence of tensile stress. The variation in residual stress as a function of the feed per tooth is described by a fourth-degree polynomial for both x-axis (4) and y-axis (5) directions:

*σ* = 1.3412(*f_z_*)^4^*k*_4_ − 23.229(*f_z_*)^3^*k*_3_ + 130.93(*f_z_*)^2^*k*_2_ − 195.82(*f_z_*)*k*_1_ + 155.23*k*_0_ (4)

*σ* = −0.0391(*f_z_*)^4^*k*_4_ + 0.6418(*f_z_*)^3^*k*_3_ − 3.5329(*f_z_*)^2^*k*_2_ + 71.673(*f_z_*)*k*_1_ − 50.286*k*_0_ (5)

where *k_i_*, representing the polynomial unit factors, corresponds to the unit ordinals:

$$k_{4}={\left[\frac{1}{mm}\right]}^{4}\cdot MPa;\;k_{3}={\left[\frac{1}{mm}\right]}^{3}\cdot MPa;\;k_{2}={\left[\frac{1}{mm}\right]}^{2}\cdot MPa;\;k_{1}=\frac{1}{mm}\cdot MPa;\;k_{0}=1\;MPa.$$

**Figure 8:**
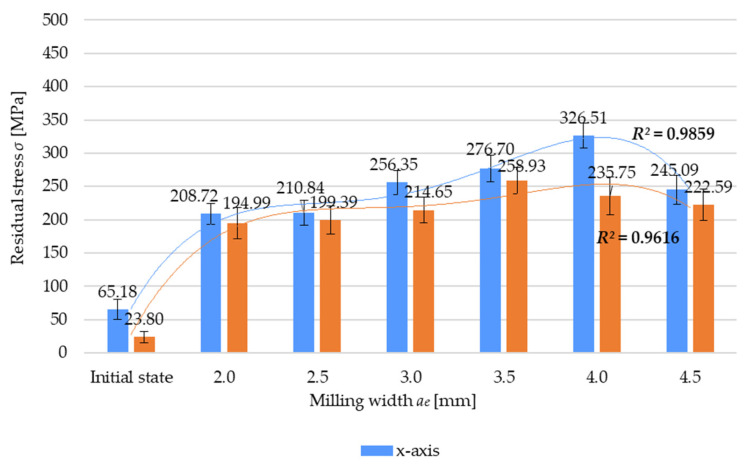
Residual stress *σ* versus milling width *a_e_*.

Figure 8 shows the residual stress *σ* as a function of the milling width *a_e_*. The results demonstrate that the maximum residual stress is obtained at *a_e_* = 4 mm for the x-axis direction and at *a_e_* = 3.5 mm for the y-axis direction. For the case with *a_e_* = 4 mm and the x-axis direction, the difference between the maximum residual stress and prestress amounts to 400% (relative to the initial state). For *a_e_* = 3.5 mm and the y-axis direction, this difference is almost 1000%. The results of residual stress and the effect of milling width show the presence of tensile residual stress in the surface layer of the material. The variation in residual stress as a function of the milling width was approximated by a fourth-degree polynomial for the results obtained for the x-axis (6) and y-axis (7) direction:

*σ* = −2.993(*a_e_*)^4^*k*_4_ + 47.785(*a_e_*)^3^*k*_3_ − 268.59(*a_e_*)^2^*k*_2_ + 652.02(*a_e_*)*k*_1_ − 361.82*k*_0_ (6)

*σ* = −2.0068(*a_e_*)^4^*k*_4_ + 34.844(*a_e_*)^3^*k*_3_ − 218.2(*a_e_*)^2^*k*_2_ + 593.97(*a_e_*)*k*_1_ − 381.11*k*_0_ (7)

where *k_i_*, representing the polynomial unit factors, corresponds to the unit ordinals:

$$k_{4}={\left[\frac{1}{mm}\right]}^{4}\cdot MPa;\;k_{3}={\left[\frac{1}{mm}\right]}^{3}\cdot MPa;\;k_{2}={\left[\frac{1}{mm}\right]}^{2}\cdot MPa;\;k_{1}=\frac{1}{mm}\cdot MPa;\;k_{0}=1\;MPa.$$

**Figure 9:**
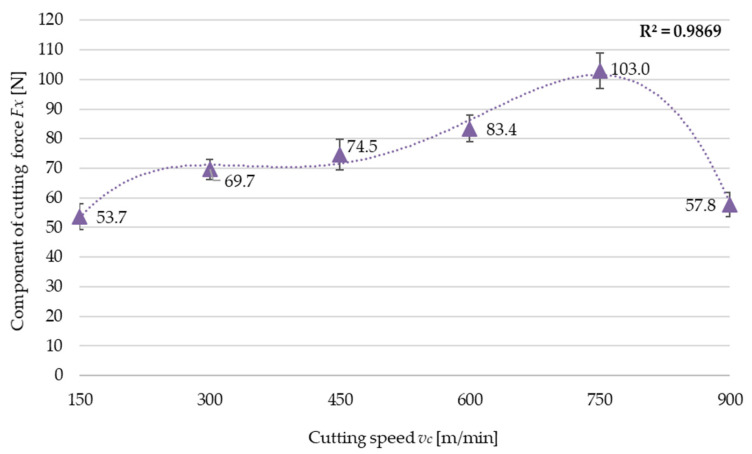
Cutting force component *Fx* versus cutting speed *v_c_*.

Results of the cutting force components are shown in Figure 9 for the *Fx* component as a function of the cutting speed *v_c_*. An analysis of the results reveals that the cutting force component *Fx* increases with the cutting speed *v_c_* only up to a certain point because when cutting speed *v_c_* = 750 m/min, the *Fx* component reaches the maximum value, and then it begins to decrease. This trend agrees with that observed for residual stress. The relationships obtained for variable feed per tooth *f_z_* and milling width *a_e_* show agreement with those obtained for residual stress. The results of the cutting force component as a function of the cutting speed are described by a fourth-degree polynomial function (8):

(8) $$Fx=-4\cdot 10^{-9}{\left(v_{c}\right)}^{4}k_{4}+7\cdot 10^{-6}{\left(v_{c}\right)}^{3}k_{3}-0.0047{\left(v_{c}\right)}^{2}k_{2}+1.3055\left(v_{c}\right)k_{1}-58.454k_{0}$$

where *k_i_*, representing the polynomial unit factors, corresponds to the unit ordinals:

$$k_{4}=1{\left[\frac{min}{m}\right]}^{4}\cdot N;\;k_{3}={1[\frac{min}{m}]}^{3}\cdot N;\;k_{2}=1{\left[\frac{min}{m}\right]}^{2}\cdot N;\;k_{1}=1\frac{min}{m}\cdot N;\;k_{0}=1\;N.$$

**Figure 10:**
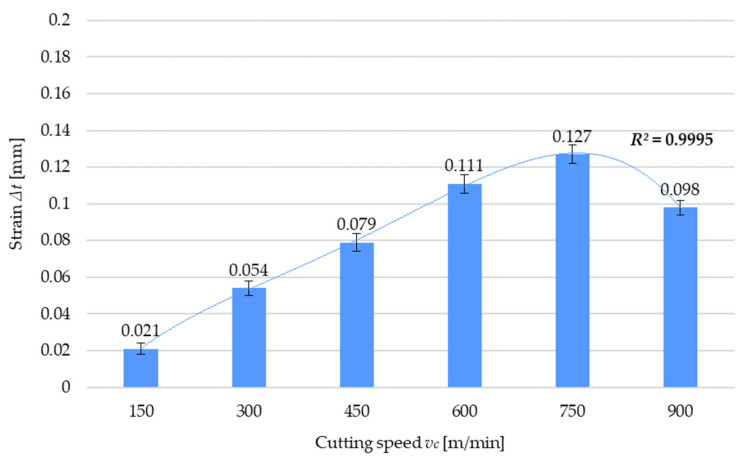
Strain Δ*t* versus cutting speed *v_c_*.

The next step of the study involved analyzing the strain of the thin wall of the sample, considering the residual stress results and, at the same time, evaluating the effect of the tested technological parameters of milling. Figure 10 shows an example of strain Δ*t* as a function of the cutting speed *v_c_*, demonstrating that the strain results agree with the results of residual stress as well as that the relationships between strain and technological parameters are the same as those established for residual stress. The results of strain as a function of cutting speed are approximated by a fourth-degree polynomial function (9):

Δ*t* = −0.0009(*v_c_*)^4^*k*_4_ + 0.0105(*v_c_*)^3^*k*_3_ − 0.0426(*v_c_*)^2^*k*_2_ + 0.1005(*v_c_*)*k*_1_ − 0.0463*k*_0_ (9)

where *k_i_*, representing the polynomial unit factors, corresponds to the unit ordinals:

$$k_{4}=1{\left[\frac{min}{m}\right]}^{4}\cdot mm;\;k_{3}={1[\frac{min}{m}]}^{3}\cdot mm;\;k_{2}=1{\left[\frac{min}{m}\right]}^{2}\cdot mm;\;k_{1}=1\frac{min}{m}\cdot mm;\;k_{0}=1\;mm.$$

Table 4 presents a comparison between the residual stress *σ* and the strain Δ*t* for the variable cutting speed *v_c_*. Both of these quantities were determined experimentally using appropriate research equipment (Figure 2). Based on the results, a correlation between the residual stress in the surface layer and strain was found. As the residual stress increases, the strain also increases. This relationship is particularly observable in the case of thin-walled elements, but for elements with large cross-sections, residual stress may have a smaller impact on the dimensional and shape accuracy of the part.

**Figure 11:**
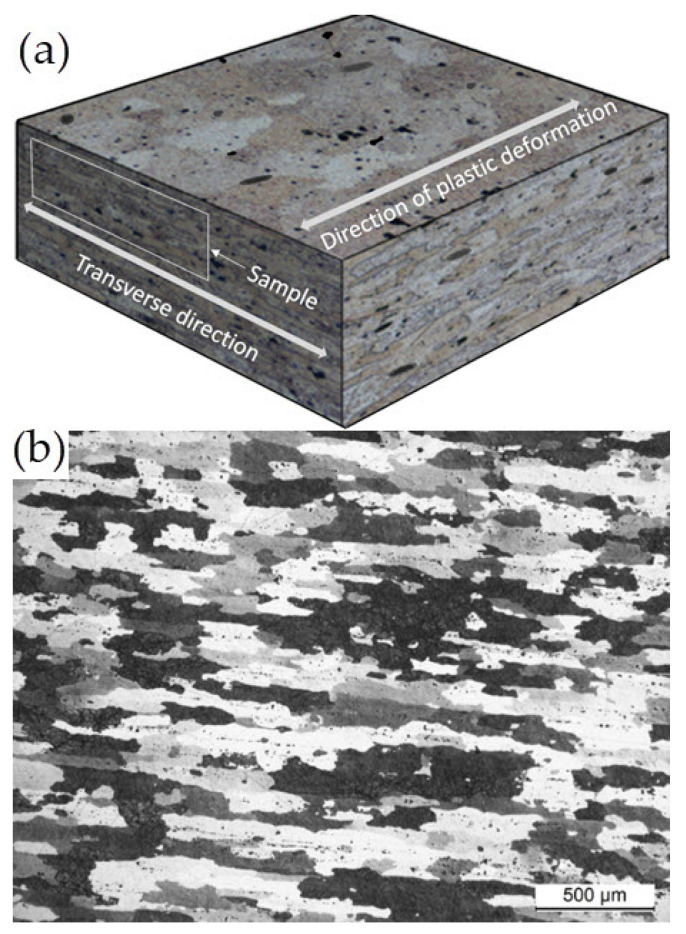
Scheme showing the location of microstructural examination and the direction of deformation of EN AW-2024 T351 in the cross-section transverse to the deformation direction: (**a**) scheme; (**b**) microstructure for 5× magnitude.

**Figure 12:**
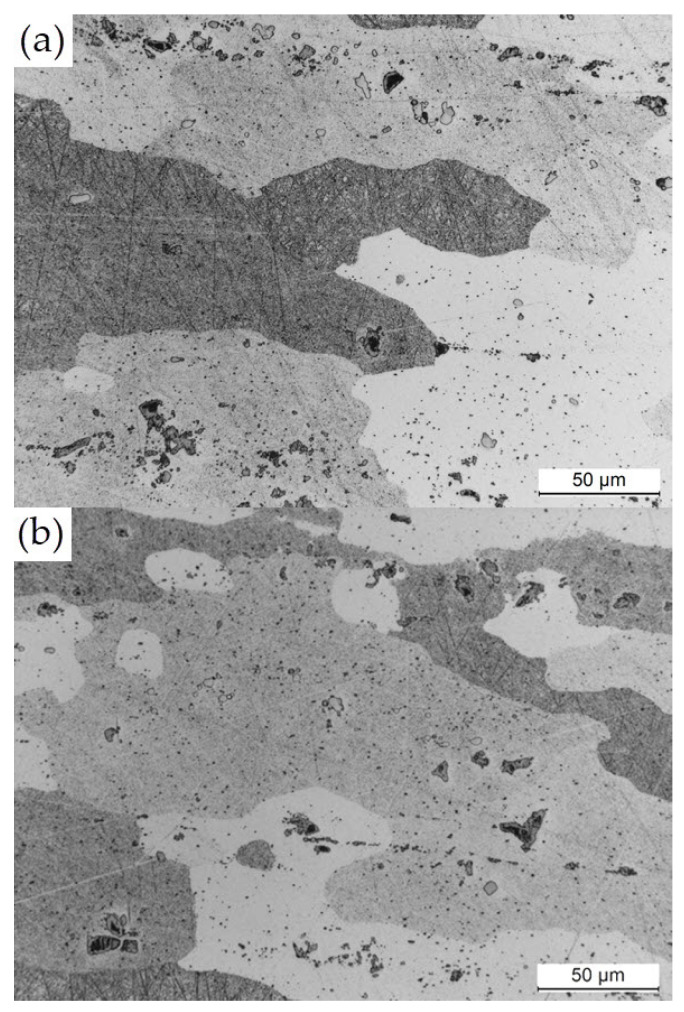
Microstructure of EN AW-2024 T351 in the cross-section transverse to the direction of deformation for 50× magnitude: (**a**) example 1; (**b**) example 2.

The examination of the cross-section of the sample transverse to the direction of deformation has shown that the microstructure of the EN AW-2024 T351 alloy is typical of deformed alloys (Figure 11a). Deformed and flattened grains are visible over the entire volume of the alloy (Figure 11b). The examination performed at higher magnification has shown the presence of primary, undissolved intermetallic phases, primarily Al_7_Cu_2_Fe and α-Al_15_(FeMn)_3_(SiCu)_2_, as well as θ-Al_2_Cu, S-Al_2_CuMg, and β-Mg_2_Si, which are fragmented due to deformation. The precipitates are either distributed inside the α-Al solid solution grains or they form a banded structure that is characteristic of the deformed state. In addition to that, very fine and homogeneously distributed precipitates of the secondary strengthening phases are visible in the α-Al solid solution (Figure 12).

Additionally, as part of the residual stress test, a statistical analysis was performed to assess the significance of the obtained differences. First, the normality of the distribution of the studied variables was checked using the Shapiro–Wilk *W* test. The results showed that for a significance level of *α* = 0.05, the studied variables had a normal distribution because Equation (10) was satisfied:

(10) W≥W(n,α)

where W(n,α) denoted the critical value of the Shapiro–Wilk *W* test, taking into account the number of measurements *n* and the assumed significance level *α*.

After that, the hypothesis of the equality of variances was verified. The Fisher–Snedecor *F* statistic was used to that end. $\sigma_{1}^{2}$ and $\sigma_{2}^{2}$ were assumed to be the variances of the variable in the first and second populations, and the following hypotheses were formulated:

- *H*_0_: $\sigma_{1}^{2}=\sigma_{2}^{2}$;
- *H*_1_: $\sigma_{1}^{2}\neq \sigma_{2}^{2}$.

The Fisher–Snedecor *F* statistic was determined from Equation (11):

(11) $$F=\frac{s_{1}^{2}}{s_{2}^{2}}$$

where $s_{1}^{2}$, $s_{2}^{2}$ were the variances of the studied variable in the samples randomly selected from the first and second populations ($s_{1}^{2}$ was the variance with a greater value than $s_{2}^{2}$).

Based on the adopted significance level *α* and the determined numbers of degrees of freedom *f*_1_ and *f*_2_, the critical value *F_cr_* was established. If Equation (12) was met, *H*_0_ was accepted:

(12) $$F<F_{cr}$$

Since the hypothesis of the equality of variances was confirmed in each case, the Student’s *t*-test was used in the next step to test the hypothesis of the equality of mean values. Two hypotheses were formulated:

- *H*_0_: $u_{1}=u_{2}$;
- *H*_1_: $u_{1}\neq u_{2}$.

The value of the *t*-test statistic was determined from Equation (13):

(13) $$t=\frac{\bar{X_{1}}-\bar{X_{2}}}{\sqrt{\frac{n_{1}S_{1}^{2}+n_{2}S_{2}^{2}}{n_{1}+n_{2}-2}(\frac{1}{n_{1}}+\frac{1}{n_{2}})}}$$

where $\bar{X_{1}}$, $\bar{X_{2}}$ were the mean values, $S_{1},S_{2}$ were the variances, and $n_{1},n_{2}$ denoted the number of samples.

The specific two-sided critical area was defined as (14):

(14) $$(-\infty ,-t_{cr}\rangle \cup \langle t_{cr},+\infty )$$

If the value of the *t* test statistic belonged to the critical area, the *H*_1_ hypothesis was accepted, and if it was outside this area, then *H*_0_ was accepted, respectively.

The results of the statistical analysis for residual stress (measured in the x-axis direction) are listed in Table 5, Table 6 and Table 7.

Given the obtained results (Table 5, Table 6 and Table 7), for the adopted significance level of *α* = 0.05, the null hypothesis *H*_0_ of the equality of variances was accepted in each case. When verifying the hypothesis about the equality of mean values, it was mostly observed that the mean values were not the same, so the alternative hypothesis *H*_1_ was accepted. The exceptions were the tests of the equality of mean values for the following configurations: initial state—*v_c_* = 150 m/min and *a_e_* = 2.0 mm—*a_e_* = 2.5 mm. Therefore, it can be concluded that for the adopted significance level of *α* = 0.05, the cutting parameters have impact on the residual stress.

## 4. Discussion

The results of this study demonstrate that residual stress depends most significantly on cutting speed. An increase in the cutting speed value up to *v_c_* = 750 m/min causes an increase in residual stress, whereas at a cutting speed *v_c_* = 900 m/min, the residual stress value decreases. The pattern of residual stress variations with an increasing cutting speed is practically the same as that observed for the cutting force, which may suggest that an interconnection exists between the two quantities. In addition to that, the cutting force increase results from a higher cutting resistance due to higher strain in the cutting zone, which, consequently, leads to higher residual stress. At higher cutting speeds that are typical of High-Speed Machining, a cutting force decrease is observed, a situation which is not clearly explained. This may be due to several phenomena occurring in the cutting zone, such as:

- Increased cutting speed results in a local increase in temperature, which reduces cutting resistance due to a decrease in the coefficient of friction;
- Increased temperature improves the plasticization of the material in the shear zone, which also leads to reduced cutting resistance;
- Reduced friction as a result of increased chip flow rate.

All these phenomena can occur simultaneously. It should be stressed that each of them causes a strain decrease in the cutting zone, and hence a decrease in residual stress.

The manner in which the cutting force values changes with increasing the cutting speed is an intrinsic characteristic of this material. Thus, it is likely that this phenomenon occurs with variable residual stress as a function of the cutting speed.

Apart from the cutting speed, feed per tooth is another parameter that has a significant impact on residual stress. Increasing the value of this parameter leads to an increase in residual stress, which results from a higher cutting resistance. A similar effect takes place when the cutting width is increased, although the effect is less intense than that observed when increasing the feed per tooth.

## 5. Conclusions

The experimental results of residual stress, the cutting force component, and postmachining strain of thin-walled parts lead to the following conclusions:

- The variable cutting speed has the most significant effect on machining-induced residual stress;
- Regarding the variable cutting speed, the pattern of residual stress variations is closely correlated with the cutting force variations;
- Reduced machining resistance under HSC conditions leads to reduced values of cutting force and residual stress, which results in the reduced strain of the thin walls;
- Given the reduction in residual stress, cutting force, and strain, it is recommended that HSC be used for machining thin-walled parts;
- Residual stress is also significantly affected by variations in the feed per tooth and milling width, so the pattern of variations observed for both cases is similar, i.e., increasing the values of these parameters leads to an increase in residual stress, which is related to higher machining resistance due to an increased cross-section of the cut layer;
- It has been noted that out of the two abovementioned parameters, residual stress depends more significantly on feed per tooth;
- In general, residual stress increases with increasing the milling width, and this may be related to a greater effect of thermal interactions for low wall thicknesses. The exception is the result obtained for the greatest milling width value, i.e., *a_e_* = 4.5 mm;
- A residual stress decrease in the lowest-thickness wall leads to reduced strain, which demonstrates the importance of the effect exerted by the induced stress on the strain of machined parts, especially thin-walled ones;
- It should be noted that tensile and compressive stress occurs simultaneously, albeit with varying intensity. In the cases under study, tensile residual stress prevailed, thus indicating the dominance of the thermal stress model.

Further research should investigate the effect of the direction of feed relative to the rolling direction on residual stress. It will therefore be necessary to verify the effects of milling parallel to the direction of deformation. Moreover, further studies should investigate the effect of the value and type of residual stress as a function of the depth of deposition as well as the influence of this distribution on the strain of thin-walled elements. 

## Figures and Tables

**Table 1:** Chemical composition and properties of EN AW-2024 T351 (own elaboration, based on [52,53]).

Chemical Composition [%]														
Si	Fe	Mg		Cu	Mn		Zn	Cr		Zr+Ti	Ti		Others	Al
≤0.5	≤0.5	1.2–1.8		3.8–4.9	0.3–0.9		≤0.25	≤0.1		≤0.2	≤0.15		≤0.15	The rest
**Properties**														
Density *ρ* [g/cm^3^]			Young’s Modulus *E* [Gpa]			Tensile Strength *R_m_* [Mpa]			Yield Strength *R*_*p*0,2_ [Mpa]			Brinell Hardness Number [HB]		
2.78			73			469			324			120		

**Table 2:** Geometric parameters of SGS 44748 (own elaboration, based on [55]).

Parameter	Value
Cutting diameter [mm]	12
Overall length [mm]	100
Length of cut [mm]	48
Shrank diameter [mm]	12
Corner radius [mm]	2
Number of flutes [-]	4

**Table 3:** Milling parameters used in experiments.

	No.	Cutting Speed *v_c_* [m/min]	Feed per Tooth *f_z_* [mm/tooth]	Milling Width *a_e_* [mm]
Case 1	1	150	0.075	4.0
	2	300		
	3	450		
	4	600		
	5	750		
	6	900		
Case 2	7	750	0.025	4.0
	8		0.050	
	9		0.075	
	10		0.100	
	11		0.125	
	12		0.150	
Case 3	13	750	0.075	2.0
	14			2.5
	15			3.0
	16			3.5
	17			4.0
	18			4.5

**Table 4:** Comparison between the residual stress *σ* and the strain Δ*t*—analysis of cutting speed *v_c_*.

Constant Parameters	Cutting Speed *v_c_* [m/min]	Residual Stress *σ* [MPa]		Strain Δ*t* [mm]
		x-Axis	y-Axis	
*f_z_* = 0.075 mm/tooth *a_e_* = 4.0 mm	150	81.39	55.25	0.021
	300	119.39	78.06	0.054
	450	230.17	100.65	0.079
	600	288.22	196.42	0.111
	750	349.63	214.54	0.127
	900	258.72	188.61	0.098

**Table 5:** Statistical analysis of the residual stress results for different cutting speeds.

Tests	*F*	*F_cr_*	Result	*t*	*t_cr_*	Result
Initial state – *v_c_* = 150 m/min	2.1943	3.1789	$\sigma_{1}^{2}=\sigma_{2}^{2}$	−1.8164	2.1009	$\mu_{1}=\mu_{2}$
*v_c_* = 150 m/min– *v_c_* = 300 m/min	1.0959	3.1789	$\sigma_{1}^{2}=\sigma_{2}^{2}$	−3.5486	2.1009	$\mu_{1}\neq \mu_{2}$
*v_c_* = 300 m/min– *v_c_* = 450 m/min	1.6545	3.1789	$\sigma_{1}^{2}=\sigma_{2}^{2}$	−8.7810	2.1009	$\mu_{1}\neq \mu_{2}$
*v_c_* = 450 m/min– *v_c_* = 600 m/min	1.5539	3.1789	$\sigma_{1}^{2}=\sigma_{2}^{2}$	−4.5462	2.1009	$\mu_{1}\neq \mu_{2}$
*v_c_* = 600 m/min– *v_c_* = 750 m/min	1.2810	3.1789	$\sigma_{1}^{2}=\sigma_{2}^{2}$	−5.0889	2.1009	$\mu_{1}\neq \mu_{2}$
*v_c_* = 750 m/min– *v_c_* = 900 m/min	2.0239	3.1789	$\sigma_{1}^{2}=\sigma_{2}^{2}$	8.2242	2.1009	$\mu_{1}\neq \mu_{2}$

**Table 6:** Statistical analysis of the residual stress results for different feeds per tooth.

Tests	*F*	*F_cr_*	Result	*t*	*t_cr_*	Result
Initial state – *f_z_* = 0.025 mm/tooth	1.5378	3.1789	$\sigma_{1}^{2}=\sigma_{2}^{2}$	−11.4161	2.1009	$\mu_{1}\neq \mu_{2}$
*f_z_* = 0.025 mm/tooth– *f_z_* = 0.050 mm/tooth	2.5632	3.1789	$\sigma_{1}^{2}=\sigma_{2}^{2}$	−7.9964	2.1009	$\mu_{1}\neq \mu_{2}$
*f_z_* = 0.050 mm/tooth– *f_z_* = 0.075 mm/tooth	1.1589	3.1789	$\sigma_{1}^{2}=\sigma_{2}^{2}$	−15.6595	2.1009	$\mu_{1}\neq \mu_{2}$
*f_z_* = 0.075 mm/tooth– *f_z_* = 0.100 mm/tooth	1.0222	3.1789	$\sigma_{1}^{2}=\sigma_{2}^{2}$	−2.9061	2.1009	$\mu_{1}\neq \mu_{2}$
*f_z_* = 0.100 mm/tooth– *f_z_* = 0.125 mm/tooth	1.5189	3.1789	$\sigma_{1}^{2}=\sigma_{2}^{2}$	−4.1081	2.1009	$\mu_{1}\neq \mu_{2}$
*f_z_* = 0.125 mm/tooth– *f_z_* = 0.150 mm/tooth	2.1100	3.1789	$\sigma_{1}^{2}=\sigma_{2}^{2}$	−3.9899	2.1009	$\mu_{1}\neq \mu_{2}$

**Table 7:** Statistical analysis of the residual stress results for different milling widths.

Tests	*F*	*F_cr_*	Result	*t*	*t_cr_*	Result
Initial state– *a_e_* = 2.0 mm	1.1294	3.1789	$\sigma_{1}^{2}=\sigma_{2}^{2}$	−19.6993	2.1009	$\mu_{1}\neq \mu_{2}$
*a_e_* = 2.0 mm– *a_e_* = 2.5 mm	1.4169	3.1789	$\sigma_{1}^{2}=\sigma_{2}^{2}$	−0.2570	2.1009	$\mu_{1}=\mu_{2}$
*a_e_* = 2.5 mm– *a_e_* = 3.0 mm	1.0986	3.1789	$\sigma_{1}^{2}=\sigma_{2}^{2}$	−5.2128	2.1009	$\mu_{1}\neq \mu_{2}$
*a_e_* = 3.0 mm– *a_e_* = 3.5 mm	1.2090	3.1789	$\sigma_{1}^{2}=\sigma_{2}^{2}$	−2.2719	2.1009	$\mu_{1}\neq \mu_{2}$
*a_e_* = 3.5 mm– *a_e_* = 4.0 mm	1.1326	3.1789	$\sigma_{1}^{2}=\sigma_{2}^{2}$	−5.4778	2.1009	$\mu_{1}\neq \mu_{2}$
*a_e_* = 4.0 mm– *a_e_* = 4.5 mm	1.3858	3.1789	$\sigma_{1}^{2}=\sigma_{2}^{2}$	8.4656	2.1009	$\mu_{1}\neq \mu_{2}$

## Data Availability

Data are contained within the article.
